# Chronic Intermittent Ethanol Administration during Adolescence Produces Sex Dependent Impairments in Behavioral Flexibility and Survivability

**DOI:** 10.3390/brainsci12050606

**Published:** 2022-05-05

**Authors:** Douglas B. Matthews, Samantha Scaletty, Sarah Trapp, Abigail Kastner, Amelia M. Schneider, Areonna Schreiber, Gillian Rossmann

**Affiliations:** Department of Psychology, University of Wisconsin—Eau Claire, Eau Claire, WI 54701, USA; scaletty.samantha@mayo.edu (S.S.); strapp1@binghamton.edu (S.T.); kastnera@musc.edu (A.K.); schn1170@umn.edu (A.M.S.); schreiber.areonna@mayo.edu (A.S.); gillianrossmann@me.com (G.R.)

**Keywords:** chronic intermittent ethanol, adolescence, aged, behavioral flexibility, learning

## Abstract

Chronic intermittent ethanol exposure during adolescence produces behavioral impairments and neurobiological changes that can last into young adulthood. One such behavioral impairment is reduced behavioral flexibility, a behavioral impairment that has been correlated with the risk for increased ethanol intake. In the current study, we investigated if chronic intermittent ethanol exposure during adolescence alters cognition, including behavioral flexibility, over a 22-month testing period. Female and male rats were treated with either 3.0 g/kg or 5.0 g/kg ethanol via gavage in a chronic intermittent fashion during adolescence and then tested every 4 to 5 months on a series of cognitive measures in the Morris water maze. Chronic intermittent ethanol selectively impaired behavioral flexibility in both female and male rats, although the pattern of results was different as a function of sex. In addition, female, but not male, rats were impaired in a short-term relearning test. Finally, male rats administered ethanol during adolescence were significantly more likely to not survive the 22-month experiment compared to female rats administered ethanol during adolescence. The current results demonstrate that adolescence is a unique period of development where chronic intermittent ethanol exposure produces long-lasting, selective cognitive impairments across the lifespan.

## 1. Introduction

Alcohol (ethanol) is one of, if not the most, used and misused drug in the world [1]. Globally, alcohol misuse was attributed to approximately 3 million deaths, and for individuals between the ages of 15 and 49, alcohol was the first-leading risk factor for death and serious bodily harm [2]. Furthermore, in the United States, over 85% of people report having consumed alcohol in their lifetime, and the majority of people report having consumed alcohol in the last month [3]. Understanding the impact of alcohol exposure is a critical public health concern.

The majority of individuals first consume alcohol during adolescence [4]. For example, almost 40% of 12- to 20- year-olds have consumed alcohol at least once during their life, while ~17% of males and ~20% of females in this age group have consumed alcohol in the past month [5]. Furthermore, adolescents are not simply consuming alcohol, but the consumption pattern is often in a dangerous binge pattern [5]. It is therefore critical to understand how binge alcohol consumption during adolescence impacts the health of individuals across the lifespan.

Research has demonstrated that in some, but not all behavioral tasks, adolescent rodents respond differently than adult or aged animals to acute ethanol exposure. For example, following acute ethanol administration, adolescent rodents are less sensitive to the reduction in social inhibition compared to adult animals [6], have less ethanol-induced ataxia than adult and aged animals [7,8,9,10] and lower levels of hypnosis [11,12,13] compared to adult and aged animals. Finally, adolescents have reduced ethanol-induced hypothermia compared to adult animals and aged animals [13,14,15]. See Matthews et al. [16] for a recent review of this material.

In addition to investigating the impact of acute ethanol in adolescent animals compared to older animals, research has also focused on investigating the long-term effects of chronic, binge-like ethanol exposure during adolescence. Chronic intermittent ethanol (CIE) during adolescence can produce significant effects, including long-lasting behavioral changes that can be observed when animals are tested as adults. For example, CIE during adolescence will increase avoidance behavior when subjects are tested as adults [17,18,19,20,21,22,23,24] and will produce tolerance to ethanol-induced ataxia and hypothermia that lasts into adulthood [10,13]. Furthermore, CIE during adolescence will block the age-related increase in hypnosis to a high dose ethanol challenge [25,26]. Due to the fact that most people begin using alcohol in a binge fashion during adolescence and this exposure pattern in animals produces long-term effects, it is critical to investigate if CIE during adolescence produces effects throughout the lifespan.

The impact of CIE during adolescence on cognition has been less well studied (see [25] for a recent review). Early studies using the Morris water maze investigated if CIE treatment during adolescence in male rats led to impaired spatial learning or reduced behavioral flexibility, that is the learning of a new response strategy when challenged with previously learned stimuli. In these studies, spatial learning in the Morris water maze was not impaired by CIE via intraperitoneal injections during adolescence when training occurred during the treatment period [26,27]. Furthermore, a three-day reversal learning task in the water maze, a common measure of behavioral flexibility, was also not significantly altered by CIE treatment [27]. However, more recent studies have demonstrated CIE during adolescence can impair behavioral flexibility with no corresponding impairment in spatial learning if the initial spatial learning occurs following the treatment period and testing occurs during early adulthood. For example, adolescent male and female rats were exposed to five cycles of ethanol vapor and then tested 36-days later (post-natal day [PND] 80) in a lever pressing task. Adolescent ethanol exposure reduced behavioral flexibility in female rats when tested as adults, but not in male rats [28], although other studies have shown both male rats and mice have impaired behavioral flexibility when previously exposed to CIE via gavage during adolescence [17,29,30]. For example, adolescent male rats exposed to ethanol chronically via vapor [31] or via self-administration [32] demonstrate impaired behavioral flexibility when tested as young adults. Finally, CIE during adolescence via gavage can impair behavioral flexibility in both adult male and female rats [33]. The previous studies suggest that CIE during adolescence produces a selective impairment in behavioral flexibility in early adulthood and is less likely to produce significant impairments in general spatial learning and/or memory in early adulthood.

The impairment in behavioral flexibility due to CIE has recently received intense investigation due to clinical research demonstrating that reduced behavioral flexibility is found in abstinent alcoholics [34]. In addition, preclinical research in male rhesus monkeys has also shown that those subjects who have lower baseline behavioral flexibility are more likely to consume higher amounts of ethanol [35]. The reduction in behavioral flexibility is thought to involve a shift from goal-directed behavior to habit-directed behavior [36,37], perhaps by altering activity in the orbital frontal cortex [38] or modifying the posterior orbital frontal cortex and associated anterior insula [39]. The increase in habitual responding, i.e., a decrease in behavioral flexibility, results in continued drug taking in the face of altered outcomes and/or negative consequences (see [40,41] for reviews on this topic). Given the potential causal effect of CIE during adolescence on decreased behavioral flexibility and increased risk for alcohol misuse later in life, additional research is needed to investigate the long-term cognitive consequences of ethanol exposure in adolescence.

Research has demonstrated that CIE during adolescence can alter behavioral and neurobiological markers several months following exposure (i.e., middle adulthood). For example, CIE during adolescence alters brain-derived neurotrophic factor (BDNF) expression at PND 135 [42], impairs novel object recognition at PND 165, and reduces hippocampal volume at PND 222 [43]. While these data demonstrate a long-term effect of CIE during adolescence on behavior, we are only aware of a single study that investigated if CIE during adolescence produces alterations in cognition into later life (PND 532) [44]. In this study, male rats received either ethanol or water during adolescence via intraperitoneal injections and behavioral data was collected every ~four months until PD 532. Interestingly, CIE during adolescence produced tolerance to a high dose ethanol challenge late in life, as measured by loss of righting reflex, but more importantly for the present work, CIE did not alter spatial memory across the lifespan, but did produce a greater impairment to a low dose ethanol challenge in a spatial memory test. However, this study had several limitations, including it did not investigate if CIE during adolescence impairs behavioral flexibility across the lifespan, and it did not investigate if female rats differed from male rats in the impact of CIE during adolescence on spatial memory and behavioral flexibility.

The current study investigates if CIE via ethanol gavage (CIEg) during adolescence produces differential impairments in spatial and non-spatial learning and memory in male and female rats. In addition, we investigate if CIEg during adolescence differentially impairs behavioral flexibility in the same animals. Animals were tested at specific time points until 22 months of age, thereby allowing for an assessment of CIEg during adolescence across a majority of the animals’ lifespan. Our results demonstrate that CIEg during adolescence selectively impairs behavioral flexibility, and the impairment is different in female rats compared to male rats.

## 2. Materials and Methods

### 2.1. Subjects

Forty-two male and forty-two female Sprague-Dawley rats (Enivgo, Indianapolis, IN, USA) were used to investigate the effect of chronic intermittent ethanol via gavage during adolescence on spatial memory, non-spatial memory, and behavioral flexibility across the lifespan. Female subjects arrived in the colony on PND 28 and two days later were randomly divided into one of three ethanol conditions (see below) for treatment during adolescence (PND 30–PND 48). Male subjects arrived in the colony on PND 28 week ending and were between the ages of PND 28 and PND 34. Similar to females, two days following arrival, the males were divided into one of three ethanol conditions (see below) for treatment during adolescence (e.g., PND 34–PND 52). Animal care procedures followed the guidelines of the University of Wisconsin–Eau Claire IACUC. Food and water were provided ad libitum, except for the water control groups during the CIEg treatment period where food access was controlled to yoke the body weight of the control groups to the body weight of the high dose ethanol group, in order to minimize any difference (see below). Animals were housed two subjects per cage in an Ecoflo system (Allentown Caging, Allentown, NJ, USA) on a 12:12 light:dark cycle (lights on at 6:00 am; lights off at 6:00 pm). All animal procedures occurred between 8 am and 2 pm on test days.

### 2.2. Chronic Intermittent Ethanol Exposure via Gavage (CIEg)

Beginning on PND 30 (females’ initial body weight = 69 g)) or ~PND 34 (males’ initial body weight = 133 g) (two days after arriving in the colony), animals were randomly divided into one of two ethanol groups or one control group and received either ethanol (3.0 g/kg (*n* = 14 for male and female) or 5.0 g/kg (*n* = 14 for male and female)) or water (water amount was matched in volume to the 5.0 g/kg ethanol amount [*n* = 14 for male and female]). Ethanol, 35% *v*/*v*, was administered via gavage every 48 h for 20 days, for a total of 10 intoxications and withdrawals. During treatment, all animals in the CIEg exposure groups had unrestricted access to food and water; all animals that received water gavage (i.e., the control group) were weight yoked only during the CIEg procedure to the average 5.0 g/kg CIE-treated rats’ weight to control for ethanol-induced weight suppression ([7] for procedure and impact of body weights during the exposure procedure). For weight yoking, control animals were fed after weighing to match the average body weight of the 5.0 g/kg ethanol group. Following CIEg all animals had unrestricted access to food and water. Animals were weighed daily as an indirect index of general health, while CIEg was ongoing and then every one to two weeks following throughout the study.

### 2.3. Blood Ethanol Concentration

Six male and three female animals underwent similar gavage CIE treatment at each of the ethanol doses to serve as BEC sentinels. For these subjects, the tail was nicked 60 min following gavage on the last treatment day and approximately 5 uL of blood was collected, centrifuged to separate the plasma, and blood ethanol concentrations were determined via an AM-1 Analox machine (Analox, North Yorkshire, UK) following manufacturing guidelines. Blood ethanol levels were within previously reported ranges [45,46] (males: 134 mg/dl [3.0 g/kg] and 165 mg/dl [5.0 g/kg]; females: 134 mg/dl [3.0 g/kg] and 149 m/dl [5.0 g/kg]).

### 2.4. Impact of CIE during Adolescence on Non-Spatial and Spatial Learning

**Figure 1:**
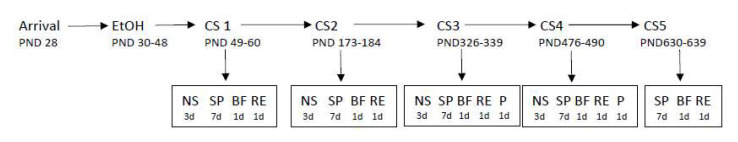
Experimental Timeline. Subjects arrived in the colony and were treated with ethanol every other day for 20 days followed by five unique cognitive testing sessions for the next 20 months. PND denotes age (post-natal day), CS denotes cognitive session, NS denotes. nonspatial learning, SP denotes spatial learning, BF denotes behavioral flexibility test, RE denotes relearning test, P denotes probe trial. The number of days of each test is listed under the specific test. See method section for specific details of each test.

All subjects underwent a non-spatial training procedure followed by a spatial training procedure and then a 1-day behavioral flexibility and a 1-day relearning assessment at specific time points following completion of the CIEg treatment. Specifically, animals first received three non-spatial training days (see below) followed by seven spatial training days (see below) before receiving two spatial reversal days (see below). In addition, the first reversal day was used to assess behavioral flexibility, while the second reversal learning day was used to assess relearning. As such, each cognitive session (CS) lasted a total of 12 days. The total cognitive session was repeated 3 additional times throughout the lifespan of the animal (CS 1–4). CS 1 began the day following completion of the CIEg treatment (PND 49 for females and ~PND 53 for males), CS 2 began 4 months later (PND 173 for females and ~PND 177 for males), CS 3 began 5 months later (PND 326 for females and ~PND 330 for males), and CS 4 began 5 months later (PND 476 for females and ~PND 480 for males). In addition, on 21 months, spatial learning, behavioral flexibility, and relearning was again assessed (CS 5) by training animals in the same testing room to a new spatial location, where the platform was never located, for seven days before an additional two-day reversal trial (no previous non-spatial training in CS 5). Finally, following the second reversal training day in CS 3 and CS 4, the platform was removed for a one-day probe trial to access spatial memory in these cognitive sessions. See Figure 1 for an experimental timeline.

### 2.5. Non-Spatial, Cue-Based, Learning (for Cognitive Sessions 1–4)

Each CS began with a three-day training in the standard non-spatial, cue-based learning procedure [47]. The tank used was six feet in diameter and the water level was approximately 24 inches deep. The tank was filled with water that was made slightly opaque with the addition of white tempera non-toxic watercolor paint. Animals were trained to swim from the four compass locations to a platform that protruded above the water surface by approximately 1.0 cm and was covered in black electrical tape to make the platform visible. Animals received four trials per day for three days. The order of the start locations was constant, while the location of the platform was changed every day to ensure animals could not use spatial cues to guide performance. The latency to the platform and swim pathlength for each animal was determined using Any-maze digital tracking software version 5.3 (Any-maze, Stoelting Co., Wood Dale, IL 60191, USA).

### 2.6. Spatial, Location-Based, Learning (for Cognitive Sessions 1–5)

For CS 1–4, spatial learning began the day following completion of the non-spatial learning, where animals were trained for seven days in the standard version of the spatial water maze task [47]. The apparatus used was the same as that used in the non-spatial, cue-based learning task. However, in this task, the escape platform was 1.0 cm below the water surface and the identifying black electrical tape was removed. In addition, the location of the escape platform was constant during training and was not one of the locations of the platform during the non-spatial training, while the order of the start locations was randomly varied. The intertrial interval was approximately 3 minutes.

Animals received four trials per day for seven days. The latency to the platform and swim pathlength was determined for each animal via Any-maze digital tracking software (Any-maze, version 5.3, Stoelting Co., Wood Dale, IL, USA). Finally, the day following the completion of the spatial training, the location of the platform was rotated 180 degrees and the animals were given two days of reversal training, four trials per day, to learn the new submerged platform location which stayed in the same location for both reversal learning days. Once again, the order of the start locations varied over the two days. The first day of reversal training reflects behavioral flexibility while the second day of reversal learning reflects short-term “relearning” of the new platform location.

For CS 5, animals were trained five months following CS 4 (subjects now post-natal 21 months of age). Animals were trained for seven days in the spatial version of the Morris water maze. For this training, the platform was located in a quadrant that had never been used for spatial training during CS 1–4. Following seven days of training, the location of the escape platform was reversed 180 degrees and behavioral flexibility was assessed for two reversal training days.

### 2.7. Probe Trials

During CS 3 and 4, twenty-four hours after the second reversal day training, the escape platform was removed, and animals were given 60 s to swim in the tank. Time in the target quadrant (the quadrant that had the platform for the reversal sessions) was monitored as a measure of spatial memory.

### 2.8. Statistical Analysis

Due to previous findings showing that male and female rats learn at different rates in the Morris water maze [48], we first investigated if a sex difference exists in our data in learning the spatial version of the Morris water maze. To investigate this, we analyzed the spatial learning of the control, water-exposed males and water-exposed females in the first spatial, hidden platform session of CS 1. As expected, a learning difference was found where males learned the spatial version of the Morris water maze task significantly faster than females, as measured by latency to the platform (Two-way ANOVA with repeated measures, sex (2) by day (7), main effect of sex, df(1,26), F = 4.612, *p* < 0.05 and main effect of Day, df(6,156), F = 17.37, *p* < 0.0001). However, the sex difference demonstrated with latency measures was not found when swim pathlength to the platform was determined (two-way ANOVA with repeated measures, sex (2) by day (7), main effect of day, df(6,156), F = 23.25, *p* < 0.0001). Importantly, the sex difference found in latency was not due to swim speed differences between males and females (Two-way ANOVA with repeated measures, sex (2) by day (7), main effect of day, df(6,156), F = 13.70, *p* < 0.0001). Data not shown. Therefore, based on the subtle, but significant difference between males and females in spatial learning as assessed by latency to the escape platform, and the slight difference in age at the start of the experiment, all future analysis were separated by sex. In addition, only data from animals that survived through the 22 months were used in analysis. This resulted in the following sample sizes: male rats had 12 control animals, 8 animals administered 3.0 g/kg ethanol and 8 animals administered 5.0 g/kg ethanol and for females there were 12 control animals, 12 animals administered 3.0 g/kg ethanol and 13 animals administered 5.0 g/kg ethanol.

Our statistical strategy was focused on investigating if chronic intermittent ethanol exposure during adolescence altered cognitive performance across the lifespan. As such, we are primarily focused on the interaction between ethanol exposure and age on cognitive function. To address this interest, we utilized ANOVA with Tukey’s post hoc tests on the percent change in cognitive function over the lifespan. Consequently, we first determined average cognitive performance of subjects in the first CS, i.e., the global performance for each animal for both latency to the platform and swim pathlength to the platform, (for both non-spatial performance and spatial performance) and then calculated percent change in performance over the next three (for non-spatial) and four (for spatial) CS that corresponded to approximately the next 20 months of the subjects’ life. For behavioral flexibility, we calculated an average score on the first reversal day, i.e., the four trials for both latency to the platform and swim pathlength to the platform, and analyzed as percent change from this score over the next four CS. Finally, for relearning, we calculated an average score on the second reversal day in CS 1, i.e., the four trials for both latency to the platform and swim pathlength to the platform and analyzed percent change for this score over the remaining cognitive sessions.

## 3. Results

### 3.1. Impact of Chronic Intermittent Ethanol Exposure on Survival

Animals were removed from the study for one of two reasons. First, subjects died by natural causes. Second, animals were removed from the study due to sudden, significant health issues, which generally included either sudden weight loss or the development of a growth/tumor that was large enough to pose a health risk to the subject.

**Figure 2:**
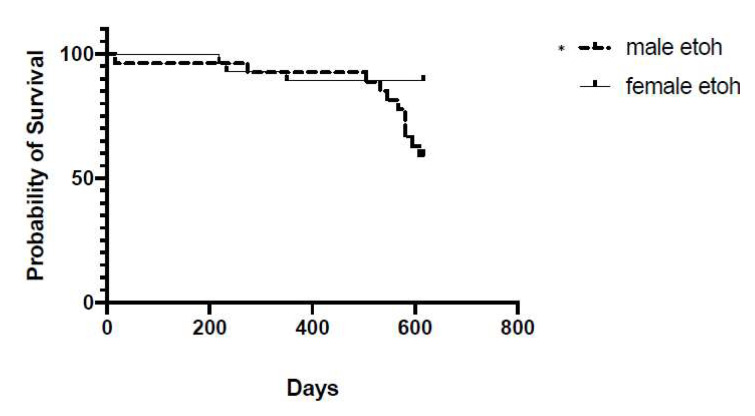
Survivability Curves Following Chronic Intermittent Ethanol. The probably of survival (y-axis) over days of the experiment (x-axis) for both males and females. As seen in the graph, males treated with alcohol were significantly more likely to not survive to the end of the experiment. * denotes significant difference, *p* = 0.017.

To investigate the impact of chronic ethanol on survivability, we first sought to ensure survivability did not differ in the water-treated animals. Specifically, we calculated survival curves and compared results using Log-Rank Mantel–Cox tests. We first determined survivability for male and female subjects that were treated with water during the adolescent treatment period to investigate if sex produced differential survival during the course of the experiment. As expected, no significant difference in survival was found between females and males that were administered water during adolescence (Log-rank Mantel–Cox test, *p* > 0.10). We next investigated if survival differences existed in males administered either 3.0 g/kg ethanol or 5.0 g/kg ethanol and no significant difference was found (Log-rank Mantel–Cox test, *p* > 0.10). We then investigated if survival differences existed in females administered either 3.0 g/kg ethanol or 5.0 g/kg ethanol and also found no significant difference on survival (Log-rank Mantel–Cox test, *p* > 0.10). Given the lack of effect on survival in both males administered ethanol and females administered ethanol, we combined the males exposed to either 3.0 g/kg or 5.0 g/kg ethanol into one group and the females exposed to 3.0 g/kg or 5.0 g/kg ethanol into another group and queried survival curves between the two sexes. Interestingly, a significant difference was found for survival following CIEg during adolescence as a function of sex. Specifically, male rats exposed to ethanol in a chronic binge-like fashion during adolescence are significantly more likely to be removed from the study (die of natural causes or euthanized due to significant health concern) than female rats exposed to ethanol in a chronic binge-like fashion during adolescents (Log-rank Mantel–Cox test, X^2^ = 5.666, *p* = 0.0173). See Figure 2.

### 3.2. Impact of Chronic Intermittent Ethanol Exposure on Bodyweight

*Males*: The average body weight of animals did not differ prior to ethanol, or water, treatment (One way ANOVA, *p* > 0.05). Similar to previous results [7], during the CIEg treatment, subjects were weighed the day of each treatment and this revealed that body weight was significantly reduced by ethanol exposure (Two-way ANOVA with repeated measures, ethanol dose by day, significant interaction of ethanol dose by day, F = 7.27, df(18,225), *p* < 0.0001). Post hoc analysis with the bodyweight of the water gavage group set as the control group, revealed that food yoking between the control group and the animals administered 5.0 g/kg ethanol was successful, in that no significant difference was found between the groups on treatment day. However, animals administered 3.0 g/kg ethanol weighed significantly more than control animals on CIEg treatment day 3 (Tukey, q = 3.16, *p* < 0.05), day 7 (Tukey, q = 4.1, *p* < 0.01), day 8 (Tukey, q = 3.3, *p* < 0.05), day 9 (Tukey, q = 3.45, *p* < 0.05) and day10 (Tukey, q = 3.6, *p* < 0.05). Further, to assess the impact of CIEg during adolescents on animals’ bodyweights, subjects were weighed either weekly or every other week (during COVID-19 restrictions), resulting in 70 different bodyweights over the course of the study. Unlike the bodyweights during the treatment, CIEg during adolescence did not significantly alter the bodyweight of animals in either the 3.0 g/kg or 5.0 g/kg condition compared to the control condition (two-way ANOVA with repeated measures, main effect of day, F = 343.2, df(69,1725), *p* < 0.0001). Data not shown.

*Females*: The average weight of animals in the different ethanol conditions (or control) did not differ at the start of the experiment (One Way ANOVA, *p* > 0.05). Similar to what was found with males during the CIEg treatment, it was found that body weight was significantly impacted by ethanol exposure (Two way ANOVA with repeated measures, ethanol dose by day, significant interaction of ethanol dose by day, F = 1.846, df(18,306), *p* < 0.0199). Post hoc analysis with the bodyweight of the water gavage group set as the control group revealed that food yoking between the control group and the animals administered 5.0 g/kg ethanol was successful, in that no significant difference was found between the groups on treatment day. However, animals administered 3.0 g/kg ethanol weighed significantly more than control animals on CIEg treatment day 7 (Tukey, q = 2.921, *p* < 0.05), day 8 (Tukey, q = 3.349, *p* < 0.01), day 9 (Tukey, q = 2.948, *p* < 0.05). Finally, and similar to what was found with males, CIEg during adolescence did not alter bodyweights after completion of the treatment and throughout the lifespan (Two way ANOVA, ethanol dose by day, main effect of day, F = 169.2, df(67,2278), *p* < 0.0001). Data not shown.

### 3.3. Impact of Chronic Intermittent Ethanol Exposure on Non-Spatial Learning

**Figure 3:**
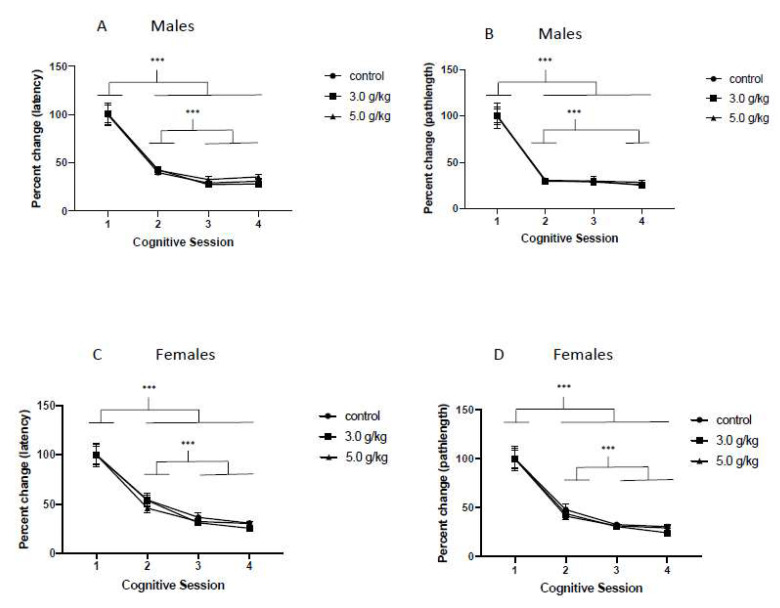
Chronic Intermittent Ethanol Exposure During Adolescence Does Not Impair Nonspatial Learning. Percent performance in the nonspatial tests for each cognitive session for males (**A**,**B**) and females (**C**,**D**) when measured by either latency to the platform (**A**,**C**) or swimpath to the platform (**B**,**D**). Brackets indicate significant post hoc tests while error bars denote standard error of the mean. *** denotes significant difference, *p* < 0.0001. A value above 100% indicates impaired performance relative to performance in CS1 while a value below 100% indicates improved performance relative to performance in CS1.

*Males*: To investigate if CIEg during adolescence impacted non-spatial learning across the lifespan, we investigated percent change in the average latency and average pathlength to the escape platform compared to performance for the first CS over the four CS (CS 1–CS 4). As expected, chronic intermittent ethanol exposure during adolescence did not alter non-spatial learning across the four CS as determined by escape latency to the platform (Two-Way ANOVA with repeated measures, ethanol dose [49] by cognitive session [50], main effect of cognitive session, F = 121.7, df(3,75), *p* < 0.0001). Tukey’s multiple post hoc test revealed that animals, regardless of ethanol dose during adolescence, performed significantly better in CS 2, CS 3, and CS 4 compared to the initial performance in CS 1 (all *p*’s < 0.0001). In addition, animals in CS 3 and CS 4 performed better than animals in CS 2 (all *p*’s < 0.0001). See Figure 3A. A similar lack of CIEg on non-spatial memory as measured by swim pathlength was also found over the first four CS (Two-Way ANOVA with repeated measures, ethanol dose [49] by cognitive session [50], main effect of cognitive session, F = 146.2, df(3,75), *p* < 0.0001). Tukey’s multiple post hoc test revealed that animals, regardless of ethanol dose during adolescence, performed significantly better in CS 2, CS 3, and CS 4 compared to the initial performance in CS 1 (all *p*’s < 0.0001). In addition, when animals were tested in CS 4, they performed better than when tested in CS 2 (*p* = 0.0034). See Figure 3B.

*Females*: Similar to what was found with males, CIEg during adolescence did not impair performance in the non-spatial memory task. Specifically, when latency to the escape platform was measured, CIEg during adolescence did not alter performance (Two way ANOVA, ethanol dose [49] by cognitive session [50], main effect of cognitive session, F = 86.09, df(3,102), *p* < 0.0001). Post hoc analysis revealed that performance was significantly better in CS 2, CS 3, and CS 4 compared to initial performance in CS 1 (Tukey’s multiple comparison test, all *p*’s < 0.0001) and performance in CS 3 and CS 4 were better than performance in CS 2 (Tukey’s multiple comparison test, all *p*’s < 0.0001). See Figure 3C. In agreement, swim pathlength to the escape platform in the non-spatial task also was not impacted by CIEg during adolescence (Two way ANOVA, ethanol dose [49] by cognitive session [50], main effect of cognitive session, F = 88.11, df (3,102), *p* < 0.0001). Post hoc analysis revealed that performance was significantly better in CS 2, CS 3, and CS 4 compared to initial performance in CS 1 (Tukey’s multiple comparison test, all *p*’s < 0.0001) and performance in CS 3 and CS 4 were better than performance in CS 2 (Tukey’s multiple comparison test, all *p*’s < 0.0001). See Figure 3D.

### 3.4. Impact of Chronic Intermittent Ethanol Exposure during Adolescence on Spatial Learning

To investigate the impact of CIEg during adolescence on spatial learning across the lifespan, we investigated the percent change in average performance in the spatial water maze task for swim latency to the submerged platform and swim pathlength to the submerged platform for each of the 5 cognitive sessions compared to performance in cognitive session 1.

**Figure 4:**
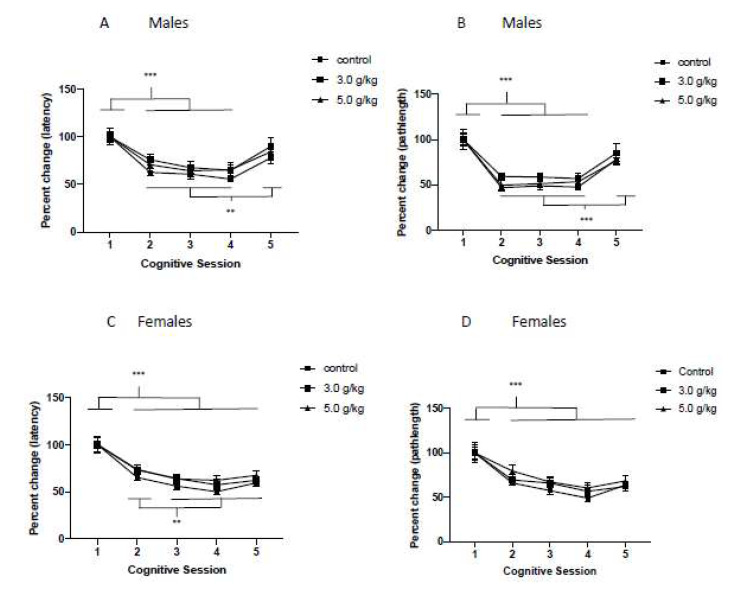
Chronic Intermittent Ethanol Exposure During Adolescence Does Not Impair Spatial Learning. Percent performance in the spatial tests for each cognitive session for males (**A**,**B**) and females (**C**,**D**) when measured by either latency to the platform (**A**,**C**) or swimpath to the platform (**B**,**D**). Brackets indicate significant post hoc tests while error bars denote standard error of the mean. *** denotes significant difference, *p* < 0.0001; ** *p* < 0.05; exact values provided in manuscript. A value above 100% indicates impaired performance relative to performance in CS1 while a value below 100% indicates improved performance relative to performance in CS1.

*Males:* Chronic intermittent ethanol exposure during adolescence did not alter spatial learning during the five cognitive sessions when measured by latency to the escape platform, even though a significant effect of age (as measured by cognitive session) of subject was found (Two way ANOVA with repeated measures, ethanol condition [49] by cognitive session [51], main effect of cognitive session, F = 20.84, df(4,100), *p* < 0.0001). Tukey’s multiple post hoc comparisons revealed that when animals were tested in CS 2, CS 3, and 4 CS they performed better than CS 1 (all *p*’s < 0.0001), indicating enhanced spatial memory during adulthood. In addition, when animals were tested in CS 5, they performed significantly worse than when they were tested in CS 2 (*p* = 0.0162), CS 3 (*p* = 0.0013), and CS 4 (*p* = 0.0003), indicating impaired spatial learning when animals were approximately 22 months of age. See Figure 4A. When spatial learning was determined by swim pathlength to the platform, an identical pattern of results emerged (Two way ANOVA with repeated measures, ethanol condition [49] by cognitive session [51], main effect of cognitive session, F = 35.45, df(4,100), *p* < 0.0001). Once again, when animals were tested in CS 2, CS 3, and CS 4, they performed significantly better than when they were tested in CS 1 (Tukey’s multiple comparison post hoc tests, all *p*’s < 0.0001) and when animals were tested in CS 5, they performed worse than in CS 2, CS 3, and CS 4 (Tukey’s multiple comparison post hoc tests, all *p*’s < 0.0001). See Figure 4B.

*Females:* Chronic intermittent ethanol exposure during adolescence did not alter spatial learning during the five CS when measured by latency to the escape platform, even though a significant effect of age of subject was found (Two way ANOVA with repeated measures, ethanol condition [49] by cognitive session [51], main effect of cognitive session, F = 47.88, df(4,136), *p* < 0.0001). Tukey’s multiple post hoc comparisons revealed that when animals were tested in CS 2, CS 3, CS 4, and CS 5, they performed better than CS 1 (all *p*’s < 0.0001), indicating enhanced spatial memory across the lifespan. In addition, compared to CS 2, animals performed better in CS 3 (*p* = 0.0348), CS 4 (*p* = 0.0007), and CS 5 (*p* = 0.0369). See Figure 4C. Chronic intermittent ethanol exposure also did not impair spatial learning when swim pathlength was analyzed (Two way ANOVA with repeated measures, ethanol condition [49] by cognitive session [51], main effect of cognitive session, F = 32.80, df(4,136), *p* < 0.0001). Tukey’s post hoc tests confirmed the findings from analysis of swim latency. Specifically, compared to CS 1, animals performed significantly better in CS 2, CS 3, CS 4, and CS 5 (all *p*’s < 0.0001). In addition, animals performed better in CS 4 compared to CS 2 (*p* = 0.0001) and CS 4 compared to CS 5 (*p* = 0.0342). See Figure 4D.

### 3.5. Impact of Chronic Intermittent Ethanol Exposure during Adolescence on Probe Trials

Chronic intermittent ethanol exposure did not alter time in the target quadrant for either males or females (all *p*’s > 0.05) in CS 3 or CS 4. Data not shown.

### 3.6. Impact of Chronic Intermittent Ethanol Exposure during Adolescence on Behavioral Flexibility

To investigate the impact of CIEg during adolescence on behavioral flexibility, we investigated the percent change on reversal day 1 for CS 1–5 compared to performance on the first day of reversal learning in CS 1 for both swim latency to the submerged platform and swim pathlength to the submerged platform (now rotated 180 degrees from the trained platform location).

**Figure 5:**
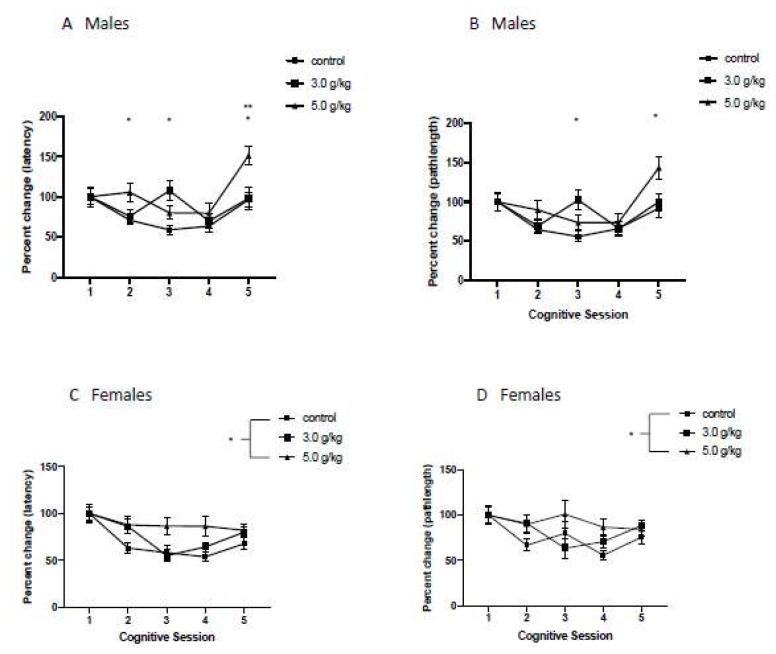
Chronic Intermittent Ethanol Exposure During Adolescence Impairs Behavioral Flexibility As a Function of Age. Percent performance on day 1 of behavioral flexibility testing for each cognitive session for males (**A**,**B**) and females (**C**,**D**) when measured by either latency to the platform (**A**,**C**) or swimpath to the platform (**B**,**D**). For males (**A**,**B**) chronic intermittent ethanol interacted with age (as determined by later cognitive sessions) to impair behavioral flexibility performance. * and ** denotes significant difference within each cognitive session, ** *p* < 0.01; * *p* < 0.05; exact values provided in manuscript. For females (**C**,**D**), animals treated with 5.0 g/kg ethanol had significantly worse behavioral flexibility compared to control animals. * *p* < 0.05; exact values provided in manuscript. Error bars denote standard error of the mean. A value above 100% indicates impaired performance relative to performance in CS1 while a value below 100% indicates improved performance relative to performance in CS1.

*Males:* Chronic intermittent ethanol exposure during adolescence produced significant impairments in behavioral flexibility, as determined by latency to the submerged platform (Two way ANOVA with repeated measures, ethanol dose [49] by cognitive session [51], significant interaction of ethanol dose and cognitive session, F = 2.873, df(8,100), *p* = 0.0064). Tukey’s multiple comparisons post hoc tests further revealed that CIEg during adolescence impacted behavioral flexibility at various timepoints across the lifespan. Specifically, animals treated with 3.0 g/kg ethanol were significantly different from control animals in CS 3 (*p* = 0.0146), while animals treated with 5.0 g/kg ethanol during adolescence performed significantly worse than control animals in CS 2 (*p* = 0.0451) and CS 5 (*p* = 0.0072). In addition, a dose dependent effect was found in CS 5, in that animals treated with 5.0 g/kg ethanol during adolescence also performed significantly worse than animals treated with 3.0 g/kg ethanol during adolescence (*p* = 0.0263). See Figure 5A.

The impact of CIEg exposure during adolescence on behavioral flexibility when analyzed with swim pathlength was also strikingly similar to that found with swim latency. Specifically, CIEg during adolescence significantly alters behavioral flexibility as measured by swim pathlength (Two way ANOVA, ethanol dose [49] by cognitive session [51], significant interaction of ethanol dose and cognitive session, F = 2.411, df(8,100), *p* = 0.0201). Tukey’s multiple post hoc comparisons confirm the impact of CIEg during adolescence on behavioral flexibility at various timepoints later in life. Specifically, subjects administered 3.0 g/kg ethanol during adolescence had impaired behavioral flexibility in CS 3 (*p* = 0.0193). In addition, animals administered 5.0 g/kg ethanol during adolescence were significantly impaired in behavioral flexibility during CS 5 compared to animals administered 3.0 g/kg ethanol during adolescence (*p* = 0.0389), and a strong trend in the data revealed an impairment compared to the control animals (*p* = 0.069). See Figure 5B.

*Females:* Chronic intermittent ethanol exposure during adolescence significantly altered behavioral flexibility across the lifespan (two-way ANOVA with repeated measures, ethanol dose [49] by cognitive session [51], main effect of ethanol dose, F = 6.96, df(2,34), *p* = 0.0029; main effect of session, F = 9.96, df(4,136), *p* < 0.0001). Tukey’s post hoc tests revealed that female animals administered 5.0 g/kg ethanol during adolescence performed significantly worse than control animals, q = 5.247, df(34), *p* = 0.0021). See Figure 5C. Due to computer error on the third behavioral flexibility test, resulting in the loss of ~20% of the swim pathlength data, we conducted a mixed factor ANOVA to account for the missing data. Once again, CIEg exposure resulted in a significant impairment in performance when swim pathlength was investigated (two-way ANOVA with repeated measures, ethanol dose [49] by cognitive session [51], main effect of ethanol dose, F = 4.193, df(2,160), *p* = 0.0168; main effect of cognitive session, F = 3.898, df(4,160), *p* = 0.0048). Similar to that found for escape latency, post hoc tests revealed that animals treated with 5.0 g/kg ethanol during adolescence resulted in worse performance compared to control animals (Tukey post hoc test, q = 4.068, *p* = 0.0126). See Figure 5D.

### 3.7. Impact of Chronic Intermittent Ethanol Exposure during Adolescence on Relearning

To investigate the impact of CIEg during adolescence on immediate relearning, we investigated the percent change on reversal day 2 for CS 1–5 compared to performance for reversal day 2 in CS 1 for both swim latency to the submerged platform and swim pathlength to the submerged platform.

**Figure 6:**
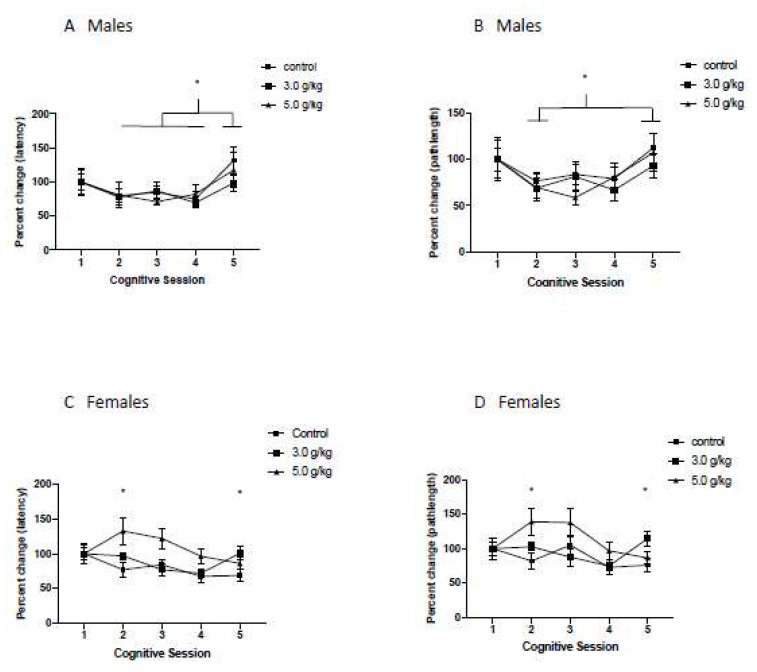
Chronic Intermittent Ethanol Exposure During Adolescence Impairs Relearning in Female Rats as a Function of Age. Percent performance of relearning (i.e., day 2 during each behavioral flexibility testing session) for each cognitive session for males (**A**,**B**) and females (**C**,**D**) when measured by either latency to the platform (**A**,**C**) or swimpath to the platform (**B**,**D**). For males (**A**,**B**) chronic intermittent ethanol did not alter relearning performance while age (as measured by increased cognitive session) did impair relearning. Brackets indicate significant post hoc tests while error bars denote standard error of the mean. * *p* < 0.05, exact values provided in manuscript. For females (**C**,**D**) chronic intermittent ethanol significantly impaired relearning due to the age of the animal. * denotes significant difference within each cognitive session, please see text for exact values. * *p* < 0.05, exact values provided in manuscript. Error bars denote standard error of the means. A value above 100% indicates impaired performance relative to performance in CS1 while a value below 100% indicates improved performance relative to performance in CS1.

*Males:* Unlike the significant impairment in behavioral flexibility performance, CIEg during adolescence did not impair relearning, as measured by either swim latency or swim pathlength to the escape platform. However, it was found that age of the animal significantly impaired relearning performance. Specifically, for swim latency to the platform, no impact of CIEg was found (two-way ANOVA with repeated measures, ethanol dose [49] by cognitive session [51], main effect of cognitive session, F = 3.611, df(4,100), *p* = 0.0166). Tukey’s post hoc analysis revealed the main effect of cognitive session was driven by a significant difference in performance between CS4 and CS5 (q = 4.162, *p* = 0.0476). See Figure 6A. A similar pattern of results was found when swim pathlength was analyzed. Specifically, no significant effect of CIEg on performance was found, but aging did significantly impair relearning (Two way ANOVA with repeated measures, ethanol dose [49] by cognitive session [51], main effect of cognitive session, F = 3.116, df(4,100), *p* = 0.0297). Tukey’s post hoc tests did not reveal particular significant differences between the different CS sessions. See Figure 6B.

*Females:* Unlike that found in male subjects, CIEg during adolescence in female subjects significantly impacted relearning performance across the lifespan for both latency to the platform and swim pathlength to the platform. Specifically, latency to the escape platform was significantly impacted by CIEg across the five CS sessions (two-way ANOVA with repeated measures, ethanol dose [49] by reversal session [51], significant interaction of ethanol dose and relearning session, F = 2.156, df(8,138), *p* = 0.0347). Post hoc tests revealed that animals administered 5.0 g/kg ethanol during adolescence had a very strong tendency toward performing worse than control animals during the relearning session of CS 2 (Tukey’s q = 3.594, *p* = 0.051) and a strong trend in the data showed impaired performance during the relearning session of CS 3 (Tukey’s q = 3.238, *p* = 0.082), while animals administered 3.0 g/kg ethanol performed significantly worse compared to control animals in reversal session of CS 5 (Tukey’s q = 3.624, *p* = 0.0452) and animals administered 5.0 g/kg in CS3 (Tukey’s q = 3.711, *p* = 0.0415). See Figure 6C. Analysis of swim pathlength confirms the impact of CIEg during adolescence on relearning across the lifespan (two-way ANOVA with repeated measures, ethanol dose [49] by relearning session [51], significant interaction of ethanol dose by reversal session, F = 2.166, df(8,136), *p* = 0.0338). Post hoc analysis once again confirmed the initial finding with swim latency. Specifically, animals administered 5.0 g/kg ethanol during adolescence performed worse during the reversal session of CS 2 (Tukey’s post hoc q = 3.583, *p* = 0.053), while animals administered 3.0 g/kg performed worse on the reversal session of CS 5 (Tukey’s post hoc q = 3.708, *p* = 0.0399). See Figure 6D.

## 4. Discussion

Chronic intermittent ethanol exposure during adolescence has been shown to produce behavioral impairments and neurobiological alterations in animal studies. The current research sought to build upon this work by investigating if CIEg during adolescence produces altered cognitive function when animals were periodically tested over a 20-month period following the ethanol exposure. We report selective, long-term cognitive deficits in animals exposed to CIEg during adolescence. First, CIEg during adolescence produced impairments in behavioral flexibility across the 20-month test period compared to performance when subjects were first tested at 2-months of age, and the pattern of impairments is different between female and male rats. Secondly, CIEg during adolescence produced sex-specific effects on relearning. Specifically, CIEg during adolescence impaired relearning in female rats compared to their initial performance, but CIEg during adolescence did not impair relearning performance in male rats. Third, the effects of CIEg on behavioral flexibility and relearning were selective in that non-spatial learning and memory performance and spatial learning and memory performance (including probe trials) were not impaired by CIEg over the 20-month test period. Finally, it was found that male rats administered CIEg during adolescence are significantly more likely to either die during the course of the study or have to be removed due to health issues compared to female rats administered CIEg during adolescence.

Behavioral flexibility is a critical cognitive construct related to understanding alcohol use disorders. Behavioral flexibility can be defined as changes in ongoing or previously learned cognitive strategies or behavioral responses due to changes in an environment, reward contingencies or internal motivational states. While numerous brain systems underly different forms of cognitive strategies, a large amount of research has shown the hippocampus and related limbic brain regions are critical in the learning of spatial tasks, or what is often called allocentric goal-directed behavior, and a second brain system is the dorsal medial striatum, which appears critical to the support of habit learning, or what was termed egocentric learning [52,53]. Successful behavioral flexibility requires a functioning interplay between goal-directed behavior and habit-directed behavior. Recently, it has been discussed that the orbital frontal cortex is critical for maintaining the successful interplay between goal-directed and habit-directed behavior to afford behavioral flexibility [41,54]. Specifically, it appears that the posterior orbital frontal cortex and associated anterior insula is critical for reversal learning [39]. This type of task requires subjects to use the opposite response strategy compared to an initial learned strategy.

Behavioral flexibility can be determined using a variety of tasks in animals including a modified Wisconsin Card Sorting task [35,55], radial arm water maze [33], foraging task [30], a working memory task in the Morris water maze [56], elevated radial arm maze [32], Barnes maze [17], and operant lever responses [28]. In the current project, we investigated behavioral flexibility using a reversal trial in the Morris water maze. Subjects were required to ignore the previously learned spatial strategy and instead search for the platform now in a new location (rotated 180 degrees from the initial trained location). Chronic intermittent ethanol exposure during adolescence impaired behavioral flexibility in both male and female rats. Specifically, in female rats, the effect existed across the experiment in animals administered 5.0 g/kg ethanol, while in males the effect was found only later in life during the last cognitive test session in animals administered the 5.0 g/kg ethanol dose.

Previous research has shown that ethanol exposure can impair behavioral flexibility. For example, long term alcohol exposure (5 months) impairs behavioral flexibility in male rats [56] while CIE during adolescence via ethanol vapor [28], gavage [17,30,33] or self-administration [32] can also impair behavioral flexibility [57]. In addition, alcohol-preferring P rats have an inherent deficit in behavioral flexibility [58]. However, only one of the previous studies investigated the effects of CIE during adolescence later in life [33] when the behavioral flexibility test occurred at approximately 4 months of age. The current project greatly extends these findings by investigating behavioral flexibility throughout the lifespan until animals are 22-months of age. By doing so, a much more complete examination of the effects of CIE during adolescence on cognitive factors has been determined. Several of these studies have found impairments in behavioral flexibility earlier in the animals’ life than reported in the current project. These differences could be due to procedure issues whereas we first trained animals in the nonspatial task then the spatial task before a behavioral flexibility test. These previous exposures in the water maze in each cognitive session may have altered performance in the behavioral flexibility test by reducing anxiety or providing practice effects in the procedure of the water maze.

In female rats, 5.0 g/kg CIEg during adolescence impaired behavioral flexibility across the 20-month exposure period compared to animals that were administered water during adolescence. While the pattern of effect in male rats was somewhat similar, the impairment in behavioral flexibility was significantly worse in male rats treated with 5.0 g/kg when animals were tested in CS 5, i.e., at approximately 22 months of age. Several possibilities exist which could lead to differential pattern of effects between male and female rats [51]. First, underlying learning performance in females compared to male rats in the Morris water maze could led to differential levels of susceptibility to CIEg on behavioral flexibility [48]. Second, ethanol has different effects in females rodents compared to male rodents, as females have greater sensitivity to the rewarding effects of ethanol and greater neurotoxicity compared to males [51,59]. Third, ethanol exposure produces differential neurosteroid levels [60] in male and female rodents. Given the neurosteroid allopregnanolone, which is elevated in rats following ethanol administration [61] can alter performance in the Morris water maze [62,63], differential increases in allopregnanolone due to ethanol exposure may alter long-term behavioral flexibility. Finally, chronic ethanol exposure impacts receptor subunit expression differentially in rodents based on sex [64] and activates microglia to a larger extent in female rats compared to male rats [49]. Further research is needed to identify factors that may underly the differential impact of sex by CIEg during adolescence on behavioral flexibility across the lifespan.

In addition to impairments in behavioral flexibility, 5.0 g/kg CIEg during adolescence in female rats significantly impaired relearning compared to the control treated female rats, an impairment that is similar to what has been previously found in male rats in an operant task [31]. This effect was sex-specific in that CIEg during adolescence did not impair relearning in male rats. The relearning data is akin to a working memory task where subjects must use the information gained on reversal day 1 of each CS (i.e., the new spatial location of the platform) to maximize performance on reversal day 2. Although previous research has shown that chronic intermittent ethanol during adolescence does not impair spatial working memory [50,65,66], it is interesting that female rats treated with CIEg during adolescence did show a relearning impairment that is similar to a working memory impairment. Although several methodical differences exist (e.g., relearning after a reversal task, repeated training in the water maze of 20 months, strain of rat, etc.), it is important to consider that female rats may be at greater risk for cognitive deficits, specifically working memory deficits, following CIEg during adolescence compared to male rats. Previous work with human subjects has shown that female adolescent drinkers have reduced working memory and lower neural activation as measured by BOLD responses in brain regions supporting working memory [67,68]. Additional research is needed to determine if brain regions underlying working memory in female rats is significantly more compromised compared to male rats by CIEg during adolescence across the lifespan.

Aged animals, compared to younger animals, are impaired in cognitive performance in the water maze. As such, it is important to determine if the cognitive impairments reported in the current work are simply due to the aging process, or, if the reported cognitive impairments are due, in part, to CIEg during adolescence. If the reported cognitive impairments were due to aging only, we would not report significant effects of either A. ethanol exposure during adolescence across the entire experiment (i.e., a significant main effect of ethanol) or B. the interaction of ethanol during adolescence and aging (i.e., a significant interaction of ethanol and cognitive session as a proxy of aging). However, this is not the case. Specifically, for nonspatial learning and spatial learning (Figure 3 and Figure 4), an age only effect is clearly present as determined by main effects of cognitive session. However, for the behavioral flexibility test (Figure 5), CIEg during adolescence significantly interacted with age in male subjects indicating that ethanol during adolescence was also impairing performance and the impairment differed by the age of the animal. This is highlighted in the last cognitive session when animals were 22 months of age. While no difference in performance due to ethanol was found for spatial learning at this time point (Figure 4), animals treated with 5.0 g/kg ethanol during adolescence performed significantly worse compared to the control and 3.0 g/kg ethanol treated animals when both latency to the platform and swim pathlength to the platform was analyzed (Figure 5A,B). Chronic intermittent ethanol exposure during adolescence also impaired behavioral flexibility performance in female rats as evidenced by a main effect of ethanol. Specifically, female rats administered 5.0 g/kg ethanol during adolescence had impaired performance that was independent of the age of the animal (Figure 5C,D). Finally, females, but not males, that were treated with CIEg during adolescence had significantly worse performance in the relearning measures (Figure 6C,D). Therefore, the reported cognitive impairments cannot be only due to the age of the animal. Instead, the cognitive impairments are at least partially due to CIEg treatment during adolescence. Furthermore, the cognitive impairments are selective in that they impair some measures of cognitive performance (behavioral flexibility and relearning) and not other measures of cognitive performance (nonspatial and spatial learning) and the selectivity may be sex-dependent.

The current project uses a longitudinal method to study the effect of CIEg during adolescence on various forms of cognition. The strength of this approach has recently been made in an exhaustive review of this topic [69]. Furthermore, we employed a repeated testing strategy in the experimental timeline. Although it is possible that carryover effects could exist which hinder meaningful conclusions, it has been argued that for many tasks, including the water maze, repeated testing in longitudinal studies is an advantage because performance is not altered due to A. stress of introducing a novel task later in the study or B. animals have already learned the procedure of the task, i.e., swim to escape or the wall of the tank is not the escape [69].

Previous research has shown behavioral flexibility to be a behavioral marker that correlates with heavy alcohol use or a previous alcohol use disorder. For example, abstinent alcoholics have impaired behavioral flexibility [34], suggesting that the inability to alter ongoing behavior (drinking) in the face of new contingencies (loss of health, work, etc.) could help explain the difficulty in overcoming alcohol misuse. However, it is unclear if the previous alcohol misuse produced the impaired behavioral flexibility or if initial low behavioral flexibility facilitated alcohol misuse. A recent study with rhesus monkeys provides interesting insights. Specifically, behavioral flexibility was assessed prior to alcohol self-administration and it was found that those subjects with low initial behavioral flexibility performance consumed more ethanol later in life than subjects with initial good behavioral flexibility [55]. This suggests that impaired behavioral flexibility correlates with the development of alcohol misuse. The current work demonstrates that female rats treated with 5.0 g/kg CIEg during adolescence have both impaired behavioral flexibility and impaired relearning. Future research should investigate if female rats administered high–dose ethanol during adolescence consume more ethanol later in life than animals not administered ethanol during adolescence. In support of this hypothesis, epidemiology data has shown that the strongest predictor of excessive alcohol use in female adults was “drunkenness-oriented” drinking during adolescence, while in males “drunkenness-oriented” drinking is the 4th strongest predictor [70].

Recently, an excellent paper reviews the effects of ethanol exposure during adolescence on later ethanol intake [71]. The majority of published studies demonstrate ethanol during adolescence will increase later ethanol self-administration in both male and female rodents. However, the pattern of results in females much more consistently demonstrates an increase in ethanol self-administration following ethanol exposure during adolescence [72,73,74,75]. Future studies should investigate if CIEg during adolescence leads to differential ethanol self-administration by sex across the lifespan. Furthermore, potential neurobiological mechanisms should be identified that may lead to high levels of ethanol drinking.

The effect of CIEg during adolescence produced selective cognitive deficits in that behavioral flexibility in both sexes and relearning in females was impaired while non-spatial learning and memory, spatial learning and memory (including spatial probe trials) were not impaired over the 20-month test session. The selectivity in cognitive impairment is important in that similar performance on most tasks following CIEg during adolescence rules out motivational differences to escape the water or motor effects from producing the impairments in behavioral flexibility and relearning. In addition, task difficulty is likely not an issue, given the inherent difficulty in spatial tasks. Finally, selective effects in several cognitive measures rules out a simple aging effect.

The prefrontal cortex and the posterior orbital frontal cortex and associated anterior insula [39] are critical for accurate behavioral flexibility. Ethanol has been shown to alter the neurophysiology of the prefrontal cortical region [76,77,78] and potentially in a sex-specific manner [79]. In addition, chronic ethanol can alter the morphology of neurons in this brain region [80]. It seems reasonable that CIEg during adolescence alters the function and perhaps structure of neurons in the prefrontal orbital frontal cortical area, thereby impacting behavioral flexibility across the lifespan. Future research needs to determine the long-term impact of CIEg during adolescence on neural function in these brain regions.

Male subjects administered ethanol in a CIEg fashion during adolescence were more likely to not survive to approximately 22 months of age compared to female rats that were administered ethanol. Animal death was due to either natural causes or removal from the study due to a developed health issue. It therefore appears that males are at a greater health risk from CIEg during adolescence than are females. These data agree with national data sets demonstrating that males are a greater percentage of alcohol related deaths compared to females [81]. However, given the small sample size in the current project (n = 14 per dose by sex at the start), we combined ethanol treated males into one group and ethanol treated females into one group for the cross-sex analysis. Future research needs to investigate the specific factors associated with survivability in animal subjects following CIEg during adolescents in larger sample sizes to more fully understand the health dynamics of CIEg during adolescence.

Several lines of active investigation have demonstrated that aged rodents are significantly more sensitive to the effects of ethanol compared to adult or adolescent animals [16]. Specifically, acute ethanol produces significantly more ataxia in aged animals [7,11,82], greater hypnosis in aged animals [11,12], larger hypothermia in aged animals [15], and greater cognitive impairments in very old animals [83] compared to younger animals. In addition, chronic ethanol may produce tolerance to a high-dose ethanol challenge faster in aged animals [84], while CIE during adolescence produces tolerance to high-dose ethanol in aged animals [44]. Given how aged animals are more sensitive to ethanol compared to younger animals, coupled with data demonstrating CIE during adolescence alters later response to the drug, future work should identify neurobiological changes in the aged brain that are impact by both acute ethanol and chronic ethanol, including CIE during adolescence.

The current research has limitations. First, we did not employ a “no-gavage” control group in an attempt to mirror similar ethanol exposure procedures using gavage from previously published work [17,30,33]. Although the addition of this control group would have assisted in determining if early stress from gavage impacted later behavior, we opted to maximize animal numbers in terms of testing two doses of ethanol. Future research should include a no-gavage condition to determine the impact of early stress on later behavioral flexibility. In addition, we opted to only determine blood ethanol levels in a subset of subjects once in the experiment to minimize additional stress on subjects. Given the extensive previous determination on blood ethanol levels after gavage in adolescent animals and the fact that the blood ethanol levels found in the current project is in line with these previously reported values, we believe this is an acceptable experimental decision. Third, we did not determine behavioral flexibility prior to ethanol administration. Future research should determine cognitive ability in individual animals prior to ethanol challenges to determine if such individual cognitive ability correlates with later ethanol effects on behavioral flexibility. Fourth, the first cognitive session occurred when animals were likely in ethanol withdrawal. However, previous work from our laboratory using a similar procedure (ethanol exposure during adolescence and Morris water maze training during this time) found no difference in learning performance due to the ethanol exposure [26,27]. In addition, we verified similar learning performance for each measure in the first cognitive session for both males and females. Therefore, it is unlikely the timing of the first cognitive session with the termination of the ethanol exposure impacted initial learning. Fifth, the age of the males and female subjects did not perfectly match. While unfortunate, ethanol exposure occurred during adolescence in both sexes, and we did not directly compare the cognitive performance of males and females. Finally, most people who begin consuming alcohol during adolescence continue to consume alcohol throughout their life. While the current project only investigates the effect of ethanol exposure during adolescence, such data will provide the framework for our future studies investigating the effect of ethanol exposure throughout the lifespan.

In conclusion, the current work provides the first data demonstrating that CIEg during adolescence produces selective cognitive deficits when subjects are tested to 22 months of age. In addition, the pattern of cognitive deficits is different between male and female subjects. Additional research is needed to identify the underlying neurobiological mechanisms producing the cognitive deficits and investigate if CIEg during adolescence produces increased ethanol intake in aged animals.

## Data Availability

The data that support the findings of this study are available from the corresponding author upon reasonable request.
